# Effects of wearing facemasks on the sensation of exertional dyspnea and exercise capacity in healthy subjects

**DOI:** 10.1371/journal.pone.0258104

**Published:** 2021-09-30

**Authors:** Isato Fukushi, Masatoshi Nakamura, Shun-ichi Kuwana

**Affiliations:** 1 Faculty of Health Sciences, Uekusa Gakuen University, Chiba, Japan; 2 Clinical Research Center, Murayama Medical Center, Musashimurayama, Japan; 3 Institute for Human Movement and Medical Sciences, Niigata University of Health and Welfare, Niigata, Japan; Ritsumeikan University, JAPAN

## Abstract

Due to the currently ongoing pandemic of coronavirus disease 2019 (COVID-19), it is strongly recommended to wear facemasks to minimize transmission risk. Wearing a facemask may have the potential to increase dyspnea and worsen cardiopulmonary parameters during exercise; however, research-based evidence is lacking. We investigated the hypothesis that wearing facemasks affects the sensation of dyspnea, pulse rate, and percutaneous arterial oxygen saturation during exercise. Healthy adults (15 men, 9 women) underwent a progressive treadmill test under 3 conditions in randomized order: wearing a surgical facemask, cloth facemask, or no facemask. Experiment was carried out once daily under each condition, for a total of 3 days. Each subject first sat on a chair for 30 minutes, then walked on a treadmill according to a Bruce protocol that was modified by us. The experiment was discontinued when the subject’s pulse rate exceeded 174 beats/min. After discontinuation, the subject immediately sat on a chair and was allowed to rest for 10 minutes. Subjects were required to rate their levels of dyspnea perception on a numerical scale. Pulse rate and percutaneous arterial oxygen saturation were continuously monitored with a pulse oximeter. These parameters were recorded in each trial every 3 minutes after the start of the exercise; the point of discontinuation; and 5 and 10 minutes after discontinuation. The following findings were obtained. Wearing a facemask does not worsen dyspnea during light to moderate exercise but worsens dyspnea during vigorous exercise. Wearing a cloth facemask increases dyspnea more than wearing a surgical facemask during exercise and increases pulse rate during vigorous exercise, but it does not increase pulse rate during less vigorous exercise. Wearing a surgical facemask does not increase pulse rate at any load level. Lastly, wearing a facemask does not affect percutaneous arterial oxygen saturation during exercise at any load level regardless of facemask type.

## Introduction

Coronavirus disease 2019 (COVID-19) is a severe acute respiratory syndrome caused by a novel coronavirus SARS-CoV-2, which has rapidly spread around the world, leading to the currently ongoing pandemic. By early December 2020, COVID-19 had ravaged the world with over 72 million total cases and more than 1.6 million deaths [1, 2]. Guidelines from the Center for Disease Control and World Health Organization recommend wearing masks to minimize the risk of transmission of COVID-19 [3, 4].

In Japan, not only patients in hospitals and people exercising in gyms but also ordinary people going about their daily activities are strictly required to wear facemasks. It is thought that wearing a facemask may increase breathing resistance and thereby augment the work of breathing, which may increase dyspnea and worsen cardiopulmonary parameters during exercise. However, there are no sufficient reports on the impact of wearing facemasks on those parameters. Therefore, in the present study, we investigated the hypothesis that wearing a facemask affects the sensation of dyspnea and physical parameters during exercise by examining the effect of wearing a surgical facemask or a cloth facemask, which are commonly used in everyday life in Japan, on dyspnea, pulse rate (PR), and percutaneous arterial oxygen saturation (SpO_2_) during exercise in healthy male adults.

## Materials and methods

### Ethical approval

**Fig 1:**
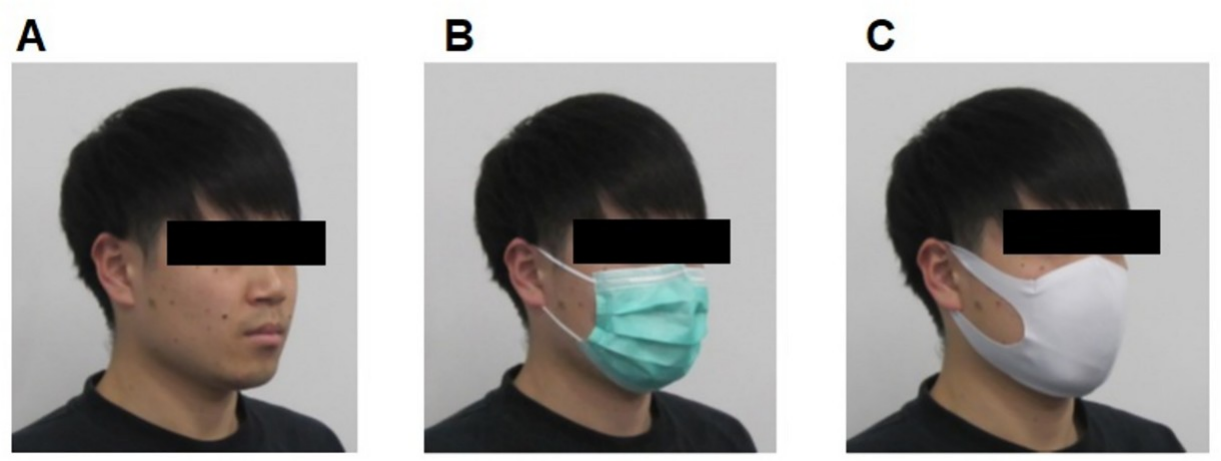
Three facemask conditions. Each participant underwent progressive treadmill tests under 3 conditions: without a facemask (A), wearing a surgical facemask (B), and wearing a cloth facemask (C) in randomized order. Both types of facemasks are widely used in hospitals and gyms as well as in daily life in Japan.

All study procedures were approved by the Uekusa Gakuen University Research Ethics Committee (approval number #20–04) and conformed to the standards set by the latest revision of the Declaration of Helsinki. This study was registered in the UMIN Clinical Trials Registry (UMIN000043230). The individual appearing in this manuscript (Fig 1) provided written informed consent to publish the potentially identifying information.

### Participants

Fifteen healthy male and 9 healthy female volunteers participated in the study. Written informed consent was obtained from all participants after providing oral and written explanations of the experimental procedures and associated risks. Participants with cardiac or pulmonary diseases or any other medical contraindications were not included. Mean demographics of the participants were as follows; age 21.0 ± 0.8 years, height 165.1 ± 7.1 cm, bodyweight 59.3 ± 7.7 kg, and body mass index 21.7 ± 2.3 kg/m^2^. Pulmonary function parameters measured with a spirometer (AS-507, Minato Medical Science, Osaka, Japan) were: vital capacity (VC), 4.0 ± 0.6 L; %VC, 92.4 ± 8.4%; forced expiratory volume in one second (FEV_1.0_), 3.4 ± 0.6 L; and %FEV_1.0_, 90.7 ± 5.9% (mean ± SD).

### Protocol

Each participant underwent progressive treadmill tests under 3 conditions: without a facemask, wearing a surgical facemask, and wearing a cloth facemask, in a randomized order. Randomization of the wearing order of facemasks was done by lottery. The participants were asked to choose a mask wearing order by picking up 1 from 6 envelopes, each of which contained 1 of 6 wearing orders. The experiment was carried out once a day under 1 of the 3 conditions, for a total of 3 days. Two types of facemasks were used: a surgical facemask (Safe+Mask^®^Premier, Medicom, Kobe, Japan) and a cloth facemask (Hadaniyasashiinunoseimask, Gunze, Tokyo, Japan), both with earloops (Fig 1). Both types of facemasks are widely used in hospitals, in gyms and in everyday life in Japan.

The PR and SpO_2_ were continuously monitored and recorded with a pulse oximeter (PULSOX Me300, Konica Minolta, Tokyo, Japan). The room temperature of the experimental laboratory was fixed at 25°C. Before commencement of the exercises, the participants were randomly assigned in the order of facemask wearing, i.e., no facemask, a cloth facemask, and a surgical facemask. First, they sat on a chair and rested for 30 minutes (stage 0). The participants then walked on a treadmill (DK-6059, Daikou, Tokyo, Japan) using a Bruce protocol that we modified: i.e., a level walking speed of 2.7 km/h with a 10% slope for 3 minutes (stage I), followed by 4.0 km/h with a 12% slope for 3 minutes (stage II), 5.5 km/h with a 14% slope for 3 minutes (stage III), and 6.8 km/h with a 16% slope for 3 minutes (stage IV). They then walked at 8.0 km/h with an 18% slope until they reached the stopping point (stage V). The exercise was discontinued when the subject’s PR exceeded 174 beats/min or when the subject wanted to stop walking. After discontinuation, the subject immediately sat on a chair and was allowed to rest for 10 minutes.

Subjects were requested to indicate the rate of perceived exertion (RPE) using a numerical rating scale. The scale ranged from 0 to 10, with “0” representing “nothing at all”, and “10” representing “very, very strong” [5]. These parameters were measured in each trial at 0, 3, 6, 9, 12, and 15 minutes after the start of measurements; at the point of discontinuation; and at 5 and 10 minutes after discontinuation.

### Statistical analysis

SPSS version 24.0 (IBM Corp., Armonk, NY, USA) was used to conduct the statistical analyses. Normal distribution of the PR and SpO_2_ data was confirmed using the Shapiro–Wilk test, skewness, kurtosis, and Q-Q plots. The data in SpO_2_ were normally distributed, but the data in PR were not. Thus, for PR, a two-way repeated analysis of variance (ANOVA) using 2 factors [time (stage 0, I, II, III, IV, and V; and recovery phase 5-minute and 10-minute) and condition (control vs. cloth facemask vs. surgical facemask)] was used to determine the interaction and main effect. Greenhouse-Geisser adjustments of the p-values were reported, since sphericity assumption was violated (p < 0.05). If the interaction effect was significant, a *post-hoc* analysis was conducted using Bonferroni’s multiple comparison test to determine differences among conditions and at each time interval. Furthermore, a Wilcoxon signed-rank sum test with Bonferroni correction was used to compare the SpO_2_ and RPE data among 3 conditions (control vs. cloth facemask vs. surgical facemask) at each time period.

Spearman’s rank correlation coefficients (r_s_) were computed to quantify the linear relationship between changes in RPE (subjective dyspnea) and changes in PR (cardiovascular system response) or SpO_2_ (objective dyspnea) between stage 0 and each time interval for each group. Statistically significant differences were assumed at p value <0.05 and descriptive data were calculated as mean ± standard deviation.

## Results

**Fig 2:**
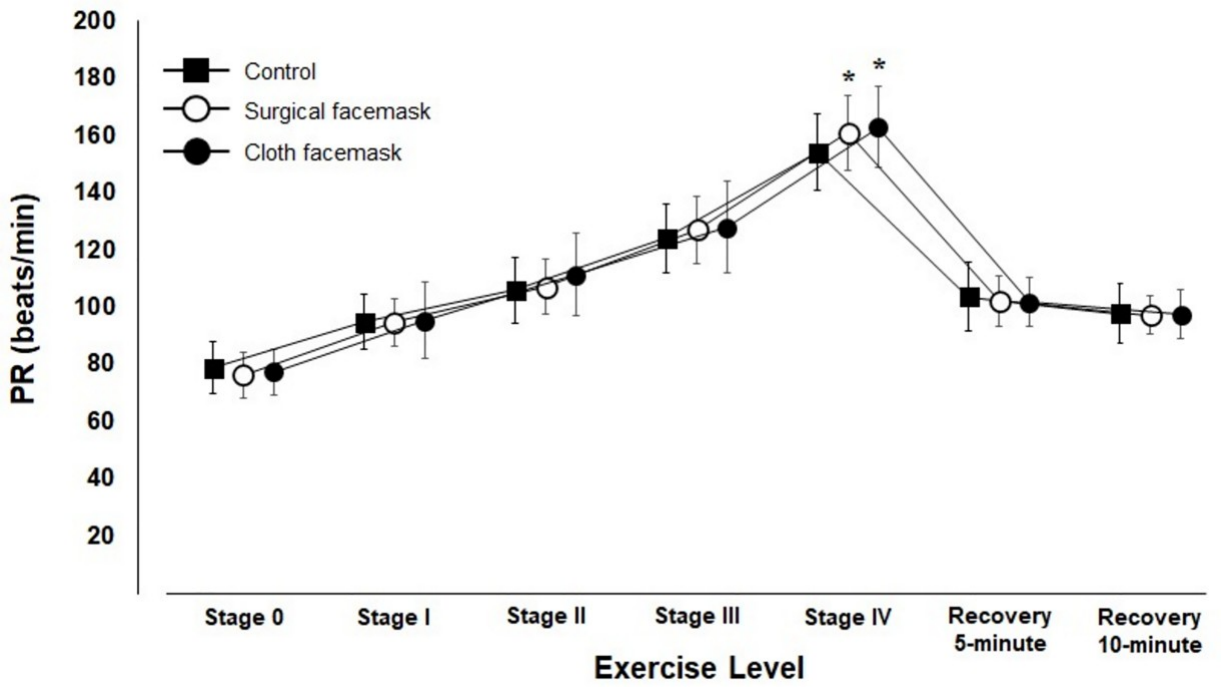
Changes in Pulse Rate (PR) before, during, and after the treadmill walking test.

Treadmill walking was discontinued when the subject’s PR exceeded 174 beats/min in all cases, but no cases was it discontinued due to a subject’s request to stop the exercise. The changes in PR values in the 3 conditions are shown in Fig 2. Two-way ANOVA showed a significant interaction effect (F = 3.06, p < 0.01, η_p_^2^ = 0.117). The Bonferroni’s multiple comparison test showed that PR values in both the cloth facemask and surgical facemask groups were higher compared with those in the control condition in only stage Ⅳ (p<0.01, and p = 0.048, respectively), but there were no significant differences in any values between the cloth facemask and surgical facemask conditions in any stages.

The SpO_2_ data are shown in Table 1. The Wilcoxon signed-rank sum test with Bonferroni correction showed that the SpO_2_ value in the surgical mask condition was significantly higher than that in the cloth facemask condition in only stage Ⅲ (p = 0.033).

**Table 1:** Changes in SpO_2_ before, during and after the treadmill walking test.

	Control	Cloth facemask	Surgical facemask
Stage 0	98 (97.75–99)	98 (97–98.25)	98 (97.75–99)
Stage Ⅰ	98 (97–98)	97 (96–98)	98 (97–99)
Stage Ⅱ	97 (97–98)	97 (96–98)	97 (97–98)
Stage Ⅲ	97 (96–97)	96.5 (96–97)	97 (96–98)*
Stage Ⅳ	96.5 (93.75–97)	96 (93.75–97)	96 (94.75–96)
Recovery 5-minute	97 (97–98)	97 (96–98)	97 (97–98)
Recovery 10-minute	98 (96–98)	97 (96.75–97)	97 (96.75–98)

*: significant difference between cloth and surgical facemask condition (p < 0.05).

The RPE data are shown in Table 2. The Wilcoxon signed-rank sum test with Bonferroni correction showed that the RPE value in the cloth facemask condition was significantly higher compared with the control condition values in stages Ⅱ, Ⅲ, and Ⅳ (p < 0.01), whereas the RPE value in the surgical facemask group was significantly higher compared with the control condition values in stages Ⅲ and Ⅳ (p < 0.05). Moreover, the PRE values in stages Ⅲ and Ⅳ in the cloth facemask condition were significantly higher than values in the surgical facemask condition (p = 0.02, and p = 0.01, respectively).

**Table 2:** Changes in Rated Perceived Exertion (RPE) scores before, during, and after the treadmill walking test.

	Control	Cloth facemask	Surgical facemask
Stage 0	0 (0–0)	0 (0–0)	0 (0–0)
Stage Ⅰ	0 (0–0.25)	0 (0–1)	0 (0–1)
Stage Ⅱ	1 (0–1.25)	1 (1–3)*	1 (1–2)
Stage Ⅲ	2 (1–3.25)	4 (3–5.25)*	3 (2–4.25) ^†^^,^ ^‡^
Stage Ⅳ	5 (3.75–6)	6.5 (6–9)*	6 (5–7.25) ^†^^,^ ^‡^
Recovery 5-minute	0 (0–1)	1 (0.75-)	1 (1–2)
Recovery 10-minute	0 (0–0)	0 (0–1)	0 (0–0.25)

Subjects were requested to indicate their RPE levels using a numerical rating scale.

*: significant difference between control and cloth facemask (p < 0.05).

†: significant difference between control and surgical facemasks (p < 0.05).

‡: significant difference between cloth facemask and surgical facemasks (p < 0.05).

Spearman’s rank correlation coefficients showed a significant correlation between the change in RPE and change in PR at stages Ⅲ and Ⅳ in the control condition (r_s_ = 0.507, p = 0.011, r_s_ = 0.436, p = 0.033, respectively), and at stage Ⅱ in the surgical facemask condition (r_s_ = 0.501, p = 0.013). Conversely, there were no significant correlation between changes in RPE and changes in SpO_2_ at any time interval in all 3 conditions.

## Discussion

We conducted experiments in which participants performed a progressive treadmill exercise under 3 conditions: without a facemask, with a surgical facemask, and with a cloth facemask, and found that wearing a facemask exacerbates dyspnea and increases PR during vigorous exercise but has no harmful effect on SpO_2_ during exercise at any load level, and that wearing a cloth facemask increased the degree of dyspnea more than wearing a surgical facemask during exercise.

During exercise, ventilation, oxygen uptake, and cardiac output are known to increase as a result of exercise. The increase in ventilation by exercise is a physiological response for the optimal maintenance of arterial blood gases and acid-base status under augmented metabolic demand conditions by muscles during exercise. Uptake of oxygen is regulated by cellular oxygen demand up to a level that equates with the maximal transport rate. Parameters that can influence the availability of oxygen include the blood’s oxygen-carrying capacity, cardiac function, peripheral blood flow redistribution, and extraction by tissues. Cardiac output is increased by exercise to meet the heightened metabolic requirements of the tissue [6]. The elevation of ventilation with increasing exercise intensity is roughly linear until reaching the ventilatory threshold of approximately 60% to 70% of the maximal exercise capacity beyond which it increases at a faster rate [7]. With low-intensity exercise, primarily oxidative fibers are recruited; however as the intensity of exercise increases, fibers that depend mainly on glycolytic pathways are recruited, leading to an increase in lactate output. The muscle fiber recruitment pattern and the potential disparity between the supply of oxygen and oxidative metabolism play a role in the rise in lactic acid with increasing exercise intensity. The lactic acid buildup lowers the pH in blood and interstitial fluid, which could ultimately lead to compromised cellular function. Subsequently, the pH reduction likely triggers ventilation as the body tries to buffer the acidity increase prompted by the decreased partial pressure of carbon dioxide. On the other hand, the uptake of oxygen and cardiac output rise in a linear fashion with workload until reaching maximal exercise capacity [6].

In our experiments, participants perceived a significantly greater sense of dyspnea when wearing a cloth facemask than when wearing no facemask, from stage II to stage IV. In addition, dyspnea was significantly greater when wearing a surgical facemask than when wearing no facemask in stages III and IV. In the present treadmill exercise test, the exercise intensity for stage II corresponds to 7 METs; that for stage III, to 10 METs; and stage IV, 14 METs [8]. Some studies have demonstrated greater dyspnea with surgical and FFP2/N95 masks compared to without masks in healthy subjects [9–11]. However, another study reported that over the course of 1 h, wearing a surgical mask at a low to moderate work rate did not increase dyspnea in healthy subjects [12], and that wearing cloth facemasks, surgical masks, and N95 masks did not significantly worsen dyspnea at any exercise intensity in healthy subjects [13, 14]. Our results support these previous studies and show that wearing a facemask does not worsen dyspnea during light to moderate exercise; however, it worsens dyspnea during vigorous exercise. Therefore, because patients with cardiopulmonary diseases will perceive dyspnea more easily than healthy subjects [15], when prescribing exercise therapy to patients wearing cloth facemasks, it should be noted that exercise of more than 7 METs may exacerbate dyspnea more than wearing no facemask. It should also be noted that even among healthy persons wearing a surgical facemask, exercise at an intensity of 10 METs or greater may worsen dyspnea more than without wearing a facemask. On the other hand, during the post-exercise recovery phase, there was no significant difference in dyspnea between conditions with and without a facemask. Our results indicate that the possibility that wearing a facemask exacerbates dyspnea need not be taken into account during the recovery phase when prescribing exercise therapy to patients.

Dyspnea has been proposed to arise as a result of disassociation or mismatch between respiratory motor output from the respiratory center and afferent information from sensed respiratory movement [16, 17]. The respiratory neural network in the lower brainstem generates respiratory motor output, which regulates the activity of respiratory muscles, and sends ascending copies of these neural respiratory output information to the limbic system and cerebral cortex as a kind of sensation that reflects the degree of respiratory effort (motor command corollary discharge) [15, 18–20]. On the other hand, the actual ventilatory motor output, which is the result of the motor command from the lower brainstem, is monitored by mechanoreceptors in the periphery, and the monitored information is transmitted to the lower brainstem, also projected to higher centers such as the limbic system and cerebral cortex, and is integrated as integrated mechanical respiratory sensation. The input from mechanoreceptors and motor command corollary discharge are then compared in higher brain centers, and a high degree of quantitative and phase mismatch or disassociation between them is perceived as dyspnea. Further, information from peripheral and central chemoreceptors is integrated at the higher brain centers and modifies the respiratory sensation. Additionally, mental status modifies the threshold and sensitivity of dyspnea [15]. According to this mismatch theory, it is possible to explain the mechanism of our results. Wearing a facemask increased respiratory resistance, which increases the mechanical work of breathing and afferent impulses from muscle spindles in respiratory muscles, enhances integrated mechanical respiratory sensation. In addition, wearing a facemask hampered the realization of the respiratory motor output as actual ventilation corresponding to the increase of the motor command. Thus, dyspnea occurred from the increased mismatch between motor command corollary discharge and integrated mechanical respiratory sensation. The psychological burden of wearing a facemask may also worsen dyspnea.

In our experiments, the sensation of dyspnea during exercise tended to increase more when wearing a cloth facemask than when wearing a surgical facemask. The pressure difference between the outside and inside of a surgical facemask used in our experiments was less than 4.0 mmH_2_O/cm^2^. If the pressure difference of a cloth facemask is greater than that of a surgical facemask, then the breathing resistance would be greater when wearing a cloth facemask than when wearing a surgical facemask, and the sense of dyspnea during exercise would be greater. However, we were not able to measure the pressure difference of a cloth mask. The underlying explanation is that cloth facemasks fit the face more tightly than surgical facemasks and the gap between the face and the facemask is smaller. The effect of cloth facemasks on breathing resistance needs to be properly measured and examined.

In the present study, the use of cloth facemasks results in a significantly increased PR in stage IV. Lässing et al reported that the use of surgical facemasks was associated with a significant increase in airflow resistance, reduced oxygen uptake, and increased heart rate during continuous exercise in young healthy men [21]. Kim et al reported that wearing filtering facepiece respirators at low moderate work rates over 1 hour significantly increased heart rate [22]. In addition, there are several reports that exercise with facemask does not significantly increase heart rate [9, 13, 14]. Our results are not consistent with these reports. Cardiac output during exercise is not affected by the moderately increased work of breathing, but may be increased at high exercise intensities (>90% of maximal oxygen uptake) and with high airflow resistance [23]. In our experiment, wearing a cloth facemask significantly increased PR at stage IV. This increased PR may be induced by a combination of high intensity exercise and increased airflow resistance, making our results dissimilar from previous reports.

In the present study, neither the use of a facemask nor the type of facemask affected SpO_2_ during exercise. Our results are in agreement with previous studies on surgical masks and N95 filtering facepiece respirators [12, 24–26]. It has also been reported that wearing a surgical facemask and wearing a cloth facemask during progressive cycle ergometer exercise has only minimal and statistically inconsistent effects on oxygen saturation in healthy subjects [13, 14]. Fikenzer et al reported that capillary PO_2_, PCO_2_, and pH did not differ significantly between surgical and FFP2/N95 facemasks at maximal load in an incremental exertion test in healthy volunteers, suggesting that alveolar ventilation or gas exchange is not significantly impacted by facemasks [9]. We have newly shown that wearing a cloth facemask also has no effect on SpO_2_ during exercise at any intensity.

Very few studies have reported the effects of mask wearing on dyspnea and physiological parameters during exercise in the elderly. Molgat-Seon et al. reported that mechanical ventilatory constraint does not affect dyspnea, heart rate, or SpO_2_ during moderate intensity exercise in healthy older men and women [27]. Men over the age of 45 were able to tolerate inspiratory resistance to up to the same extent as younger men, indicating that the acceptable level of breathing resistance such as a facemask tolerable for younger men can also be applied to middle aged men [28]. However, the findings in the present study cannot be straightforwardly extrapolated to older persons and patients with pulmonary or cardiovascular diseases. For young, healthy subjects, aerobic exercise at any level of intensity with either a surgical facemask or cloth facemask may be safe and feasible. In particular, young healthy persons will not have to consider the effect of wearing a facemask on SpO_2_ even during vigorous exercise. However, in elderly persons and patients with pulmonary or cardiovascular diseases, cardiopulmonary function is lower than in young healthy subjects, and their sensitivity of dyspnea perception during exercise also differs from that of young healthy subjects. Therefore, further studies are needed regarding the effect of facemasks on dyspnea in elderly persons and patients.

The Bruce protocol is widely used around the world, and many of the published data involving treadmill exercise tests are based on the Bruce protocol [29]. However, according to the Bruce protocol, which we modified, the cumulative influence from light to moderate exercise cannot be excluded when evaluating the impact of vigorous exercise. This is a limitation of our current study. In addition, cardiac function is one of the important factors in the occurrence of dyspnea and the determination of dyspnea levels [30, 31]. The fact that we did not measure cardiac function, including blood pressure, during exercise is also a limitation of this study.

We conclude that wearing a facemask does not worsen dyspnea during light to moderate exercise but worsens dyspnea during vigorous exercise. Wearing a cloth facemask increases dyspnea more than wearing a surgical facemask during exercise. Wearing a cloth facemask increases PR during vigorous exercise, but it does not increase PR during less vigorous exercise. Wearing a surgical mask does not increase PR during any load level. Wearing a facemask does not affect SpO_2_ during exercise at any load level regardless of the facemask type. The worsening of dyspnea with facemask wearing is not necessarily due to the lowering of arterial oxygen levels, which indicated a dissociation of the respiratory sensation and pulmonary gas exchange function [15].

## Supporting information

S1 Data (XLSX)

## Abbreviations

COVID-19: Coronavirus disease 2019
FEV_1.0_: forced expiratory volume in one second
PR: pulse rate
RPE: rated perceived exertion
SpO_2_: percutaneous arterial oxygen saturation
VC: vital capacity
